# Symptom profiling for infectious intestinal disease (IID): Do symptom profiles alter with age?

**DOI:** 10.1371/journal.pone.0269676

**Published:** 2022-06-30

**Authors:** Anna L. Donaldson, John P. Harris, Roberto Vivancos, Sarah J. O’Brien

**Affiliations:** 1 NIHR Health Protection Research Unit in Gastrointestinal Infections, University of Liverpool, Liverpool, United Kingdom; 2 Institute of Population Health, University of Liverpool, Liverpool, United Kingdom; 3 National Infection Service: Field Epidemiology, Public Health England, Liverpool, United Kingdom; University of Nevada Reno School of Medicine, UNITED STATES

## Abstract

Symptom profiles have previously been identified for infectious intestinal disease (IID) which distinguish bacterial from viral organisms. However, there is evidence that the seasonality, severity, and duration of IID may differ between children, adults and elderly. A secondary data analysis was undertaken to explore whether symptom profiles for bacterial and viral IID vary across different age groups. Data from 844 cases of IID were divided into three age categories: <16 years, 16–65 years and >65 years. Multivariable logistic regression modelling was used to compare the significance of different symptoms across the three age groups. The odds of bacterial IID in children were increased by onset in the summer, diarrhoea in the absence of vomiting and fever. These symptoms were also associated with lower odds of a viral pathogen. In adults, diarrhoea but no vomiting, bloody diarrhoea and diarrhoea lasting more than 3 days were associated with increased odds of a bacterial organism, whilst onset in the winter or spring and a loss of appetite were associated with viral IID. In the elderly, diarrhoea in the absence of vomiting and diarrhoea lasting more than 3 days were associated with higher odds of bacterial IID and lower odds of a viral cause. Only diarrhoea in the absence of vomiting emerged as a key symptom across all three age groups. Variation in symptom profiles by age has implications for clinicians, public health specialists and epidemiologists who use symptoms to guide presumptive diagnoses in the absence of microbiological confirmation.

## Introduction

Infectious intestinal disease (IID) is characterised by the syndrome of diarrhoea and vomiting, in the absence of a known non-infectious cause [1]. Microbiological testing is key to providing a definitive diagnosis and yet laboratory tests identify a causative organism in less than half of samples [1, 2]. Factors such as sample volume, the age and sex of the patient and the timing of the sample all affect the likelihood of identifying an organism [1, 3]. Prior to the widespread availability of laboratory testing, symptoms were used to distinguish between bacterial and viral IID outbreaks [4–6]. These included the diarrhoea-to-vomiting ratio, fever-to-vomiting ratio, and the average duration of illness. The most notable of these, the Kaplan criteria, has subsequently been re-assessed alongside diagnostic testing and was found to be highly specific and moderately sensitive at detecting norovirus outbreaks [7]. Analysis of symptoms from a large community cohort study of IID also demonstrated that symptoms differ between bacterial and viral organisms and that symptoms may be used to dissociate bacterial and viral causes of IID in the absence of microbiological confirmation [8]. The use of IID symptoms to distinguish between organisms has particular relevance to syndromic surveillance systems, many of which use the broad syndrome of diarrhoea and vomiting to monitor and detect seasonal peaks of IID [9, 10]. Being able to distinguish between bacterial and viral IID based on symptoms alone could enhance syndromic surveillance systems and improve the detection and monitoring of these infections.

However, it should be considered whether the symptoms of IID vary with age. The severity of norovirus is known to alter across age groups, with the very young and elderly more likely to experience severe norovirus disease and require hospitalisation [11, 12]. Young children and the elderly are also more likely to experience prolonged diarrhoea with norovirus infection, and prolonged viral shedding [13–16]. Rotavirus infection, prior to vaccination, was more severe amongst young children, with older children and adults typically experiencing milder or asymptomatic infection [17]. However, adults may still be hospitalised with rotavirus infection, especially older adults and the immunosuppressed [18]. There is also evidence that the seasonality of infections may vary between children and adults, with some studies finding that cases of rotavirus and norovirus in adults occurred later in the season than cases in children [19, 20]. This study uses data from the Second Study of Infectious Intestinal Disease in the Community to explore whether symptom profiles for bacterial and viral IID vary across age groups.

## Methods

### Data sources

Data were used from the Second Study of Infectious Intestinal Disease in the Community (IID2 Study), which is described in detail elsewhere [1, 21]. For this analysis, data were included from the two main components of the IID2 study: the General Practice (GP) presentation study, which was a 12 month prospective study of people consulting a GP with symptoms of IID; and the prospective population-based cohort study, which involved weekly follow up of healthy volunteers in the community to identify any symptoms of IID. The case definition for IID was loose stools or clinically significant vomiting lasting less than 2 weeks, in the absence of a known non-infectious cause. Both studies utilised symptom questionnaires and stool sample testing of symptomatic people who met the case definition. Cases were included in this analysis if they had completed a symptom questionnaire and submitted a stool sample. Cases with negative stool samples, where no pathogen was identified, were excluded. Data from dual and triple infections were included multiple times; once for each organism identified, as the primary cause of symptoms could not be determined. All data were fully anonymised prior to inclusion in this study.

The IID2 Study received a favourable ethics opinion from the North West Research Ethics Committee (07/MRE08/5), and 37 NHS Research Management and Governance organisations for the 88 included General Practices approved the study. All participants of the IID2 Study provided written informed consent, including for the use of their information, suitably anonymised, for future research. All data from the IID2 Study were fully anonymised prior to inclusion in this study.

### Data analysis

Cases were stratified by age and divided into three categories; children under 16 years, adults aged 16–65 years, and elderly adults aged over 65 years. The outcome variable was the presence of a bacterial or viral organism in the stool sample. The number of protozoal infections were too few to allow separate analysis, but these infections were included in the comparison group for both bacterial and viral models. The explanatory variables were based on the symptoms outlined in the IID2 study symptom questionnaire, as well as the date of symptom onset (Table 1). Descriptive statistics were used to examine the proportion of reported symptoms in each age group for both bacterial and viral IID, and medians and interquartile ranges (IQR) were used to investigate differences in the duration of symptoms. Time trends were plotted to explore differences in seasonality based on age and cause. Multivariable logistic regression modelling was used to compare the significance of different symptoms across the three age groups. Continuous data were categorised prior to inclusion in the regression models (Table 1).

**Table 1:** Explanatory variables included in the analysis and their coding.

Explanatory variables	Coding
**Symptoms from IID2 questionnaire** ^a^	
Diarrhoea days	>3 days = 1 < = 3 days = 0
Bloody diarrhoea	Yes = 1 No = 0
Vomiting days	>3 days = 1 < = 3 days = 0
Nausea	Yes = 1 No = 0
Nausea days	>3 days = 1 < = 3 days = 0
Abdominal pain	Yes = 1 No = 0
Loss of appetite	Yes = 1 No = 0
Fever	Yes = 1 No = 0
Headache	Yes = 1 No = 0
Cough/nose/throat	Yes = 1 No = 0
**Combined symptom variables**	
Diarrhoea but no vomiting	Yes = 1 No = 0
Vomiting but no diarrhoea	Yes = 1 No = 0
**Additional variables**	
Date of onset ^b^	Autumn(0), Winter(1), Spring(2), Summer(3) ^c^

^a^ ‘Not sure’ responses from the original questionnaires were left blank and treated as missing data

^b^ Coded as a factor for analysis

^c^ Seasons defined by meteorological calendar

Statistical analysis was undertaken in R 4.0.4 [22]. Odds ratios (OR) were calculated using binomial backward stepwise regression and 95% confidence intervals (CI) were calculated around each estimate. Models were selected based on the Akaike information criterion (AIC). Phi coefficients were used to identify any significant correlations between the explanatory variables which might lead to mathematical problems with model fitting and variance inflation factors (VIF) were used to check the models for multicollinearity.

## Results

There was a total of 844 cases which met the case definition and inclusion criteria for this analysis. Cases ranged in age from 0 to 91 years. Approximately 35% of cases were children aged 16 or under, 46% were adults aged 16–65 years and 18% were elderly adults aged over 65 years. Over 80% of cases in children were caused by viruses compared to 62% in people aged 16–65 years and 60% of cases aged over 65 years (Table 2).

**Table 2:** The number and proportion of isolated organisms, by age group.

Pathogen	Age group—number (%)			Total
	<16yrs	16-65yrs	>65yrs	
**Bacteria**	**42 (14.1%)**	**134 (34.2%)**	**62 (40.3%)**	**238 (28.2%)**
*C*. *difficile*	0 (0.0%)	6 (1.5%)	5 (3.2%)	11 (1.3%)
*C*. *perfringens*	2 (0.7%)	10 (2.6%)	13 (8.4%)	25 (3.0%)
*Campylobacter* sp.	25 (8.4%)	93 (23.7%)	32 (20.8%)	150 (17.8%)
*E*.*coli* VTEC	1 (0.3%)	7 (1.8%)	7 (4.5%)	15 (1.8%)
Enteroaggregative *E*.*coli*	11 (3.7%)	13 (3.3%)	3 (1.9%)	27 (3.2%)
*Salmonella* sp.	2 (0.7%)	5 (1.3%)	2 (1.3%)	9 (1.1%)
*Yersinia* sp.	1 (0.3%)	0 (0.0%)	0 (0.0%)	1 (0.1%)
**Viruses**	**241 (80.9%)**	**243 (62.0%)**	**92 (59.7%)**	**576 (68.2%)**
Adenovirus	28 (9.4%)	23 (5.9%)	7 (4.5%)	58 (6.9%)
Astrovirus	23 (7.7%)	8 (2.0%)	5 (3.2%)	36 (4.3%)
Norovirus	78 (26.2%)	119 (30.4%)	40 (26.0%)	237 (28.1%)
Rotavirus	55 (18.5%)	30 (7.7%)	11 (7.1%)	96 (11.4%)
Sapovirus	57 (19.1%)	63 (16.1%)	29 (18.8%)	149 (17.7%)
**Protozoa**	**15 (5.0%)**	**15 (3.8%)**	**0 (0.0%)**	**30 (3.6%)**
*Cryptosporidium* sp.	12 (4.0%)	3 (0.8%)	0 (0.0%)	15 (1.8%)
*Giardia* sp.	3 (1.0%)	12 (3.1%)	0 (0.0%)	15 (1.8%)
**Total organisms identified**	**298**	**392**	**154**	**844**

**Fig 1:**
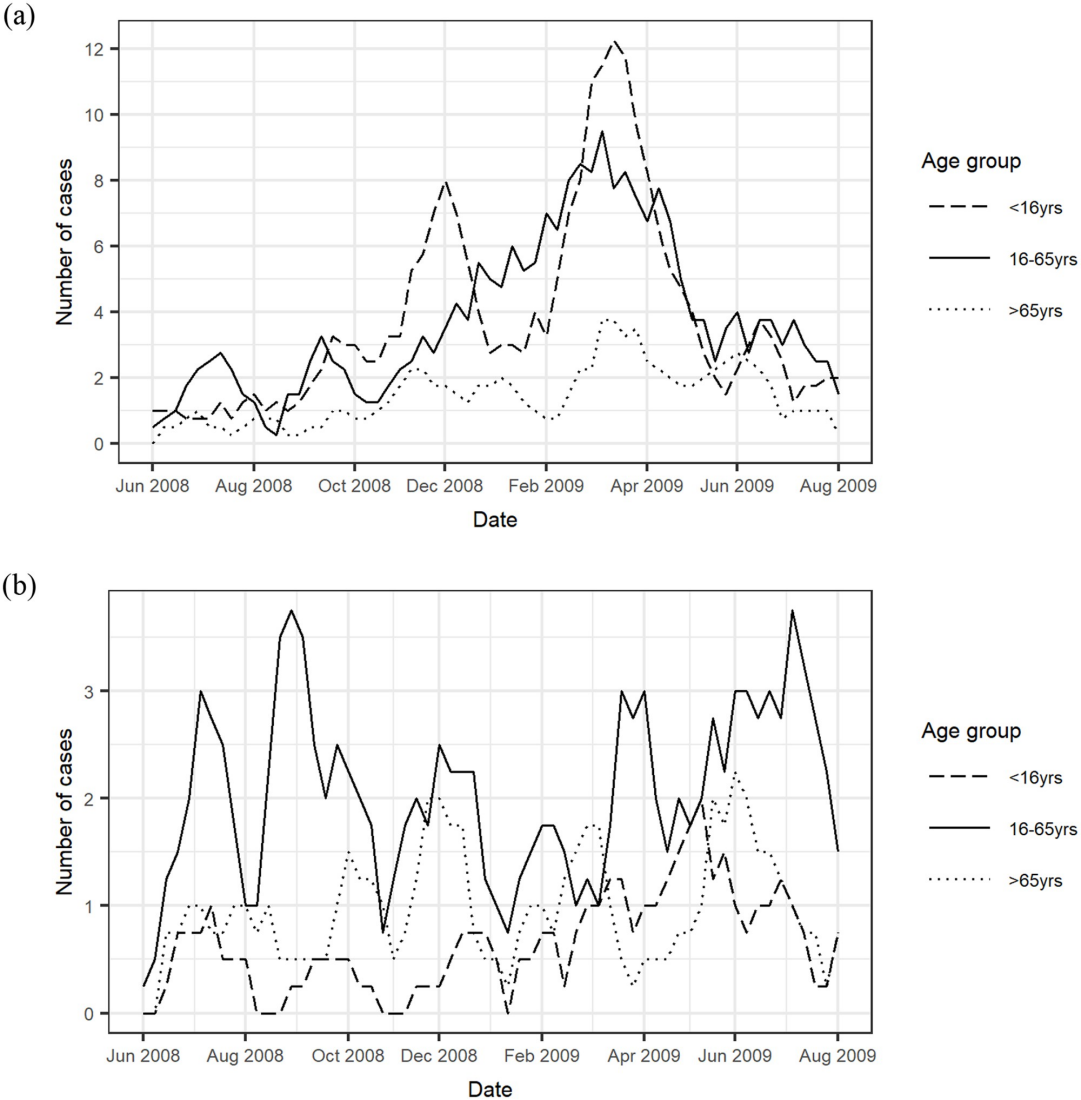
Time trends for IID, by age, for a) viral IID and b) bacterial IID, based on 4-week rolling average case numbers.

Seasonal trends differed between viral and bacterial IID, and between age groups, as shown in Fig 1. Cases of viral IID in children started increasing in September and demonstrated two peaks; one in December and a second, larger peak in March. In contrast, infections in adults increased more steadily and consistently from October to result in a single peak in March. Cases in elderly (>65 years) were lower than children and adults throughout the year and showed only a small peak in March. The number of viral IID infections were lowest for all ages in the summer months. Bacterial IID showed a less clear seasonal trend with lower case numbers than viral IID. Adults aged 16–65 years had the greatest number of bacterial IID infections, with case numbers highest over the summer months and lowest during winter. Children also showed an increase in cases over the warmer months, although case numbers remained lower than adults throughout the year. In contrast to other age groups, a summer peak was not distinguishable amongst elderly adults (>65 years).

The proportion of reported symptoms also differed across age groups (Table 3). More children experienced vomiting without diarrhoea and, for viral IID, more reported prolonged diarrhoea and vomiting, compared to adults. Of note, this was not the case for bacterial IID, where prolonged diarrhoea was less prevalent amongst children and more common in adult age groups. Adults aged 16–65 years reported more bloody diarrhoea with bacterial IID than other age groups, with 13.4% experiencing this symptom compared to 9.7% in over 65s, and 0% in children. Adults also reported more nausea, abdominal pain, and headache. Elderly adults (>65 years) reported more episodes of prolonged diarrhoea with bacterial IID, with 67.7% of bacterial diarrhoea persisting for more than 3 days, but fever was a less common symptom in this age group compared to both children and younger adults.

**Table 3:** Number and percentage of reported symptoms, by age and cause.

Explanatory variables	Children <16yrs		Adults 16-65yrs		Elderly >65yrs	
	Viral n (%)	Bacterial n (%)	Viral n (%)	Bacterial n (%)	Viral n (%)	Bacterial n (%)
Diarrhoea, no vomiting	45 (18.7%)	19 (45.2%)	86 (35.4%)	100 (74.6%)	35 (38.0%)	45 (72.6%)
Diarrhoea >3days	108 (44.8%)	20 (47.6%)	45 (18.5%)	78 (58.2%)	32 (34.8%)	42 (67.7%)
Bloody diarrhoea	2 (0.8%)	0 (0.0%)	4 (1.6%)	18 (13.4%)	1 (1.1%)	6 (9.7%)
Vomiting, no diarrhoea	34 (14.1%)	4 (9.5%)	20 (8.2%)	1 (0.7%)	2 (2.2%)	0 (0.0%)
Vomiting >3days	49 (20.3%)	3 (7.1%)	7 (2.9%)	4 (3.0%)	2 (2.2%)	2 (3.2%)
Nausea	119 (49.4%)	16 (38.1%)	195 (80.2%)	85 (63.4%)	64 (69.6%)	30 (48.4%)
Nausea >3days	38 (15.8%)	6 (14.3%)	46 (18.9%)	36 (26.9%)	21 (22.8%)	9 (14.5%)
Abdominal pain	124 (51.5%)	27 (64.3%)	178 (73.3%)	113 (84.3%)	56 (60.9%)	44 (71.0%)
Loss of appetite	197 (81.7%)	30 (71.4%)	216 (88.9%)	107 (79.9%)	81 (88.0%)	46 (74.2%)
Fever	110 (45.6%)	25 (59.5%)	93 (38.3%)	69 (51.5%)	25 (27.2%)	19 (30.6%)
Headache	39 (16.2%)	16 (38.1%)	133 (54.7%)	76 (56.7%)	40 (43.5%)	25 (40.3%)
Cough, nose and throat	131 (54.4%)	21 (50.0%)	64 (26.3%)	29 (21.6%)	19 (20.7%)	15 (24.2%)
**Total number of cases**	**241 (80.9%)**	**42 (14.1%)**	**243 (62.0%)**	**134 (34.2%)**	**92 (59.7%)**	**62 (40.3%)**

**Fig 2:**
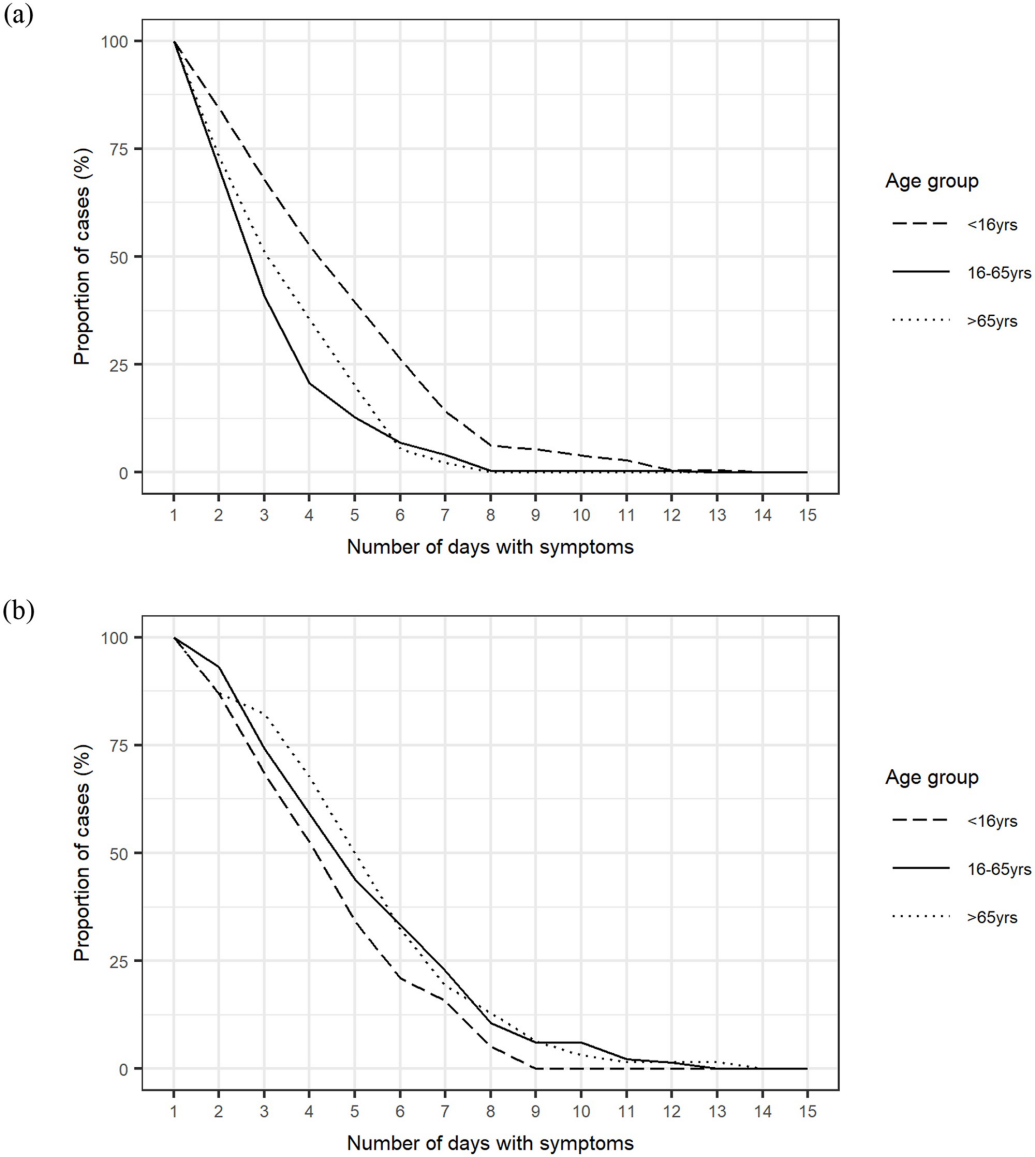
Duration of diarrhoea by age group for a) viral IID and b) bacterial IID.

The duration of diarrhoea in different age groups is shown in Fig 2. In contrast to adult age groups, children experienced more prolonged diarrhoea with viral pathogens, with a median duration of 4 days (IQR 2–6 days) compared to 2 days (IQR 1–3 days) and 3 days (IQR 1–4 days) for adults and elderly respectively, although this difference was not statistically significant. All age groups experienced a similar duration of bacterial diarrhoea, with a median duration of 4 days (IQR 2–5 days) for children, 4 days (IQR 2–6 days) for adults and 4.5 days (IQR 3–6 days) for elderly. Consequently, whilst both adult age groups showed a shorter median duration of diarrhoea with viral IID compared to bacterial, for children the duration of diarrhoea was comparable for both bacterial and viral organisms, and after 7 days 16% with bacterial IID still experienced diarrhoea, as did 14% with viral IID.

The duration of vomiting was shorter than for diarrhoea in all age groups, with median durations of 1–2 days for both bacterial and viral IID. Children had a longer duration of vomiting than the elderly age group (2 days compared to 1 day) for both bacterial and viral pathogens, although these were not statistically significant differences. The duration of nausea was similar across age groups and between bacterial and viral IID, with median durations of 2.5–3 days for bacterial organisms and 2–2.5 days for viral organisms.

### Regression modelling

In children, the odds of disease caused by a bacterial organism were higher with diarrhoea in the absence of vomiting (OR 6.69, 95% CI 2.17–22.21), fever (OR 4.84, 95% CI 1.60–17.39) and onset in the summer months (OR 4.37, 95% CI 1.13–20.07). These symptoms were also associated with lower odds of a viral pathogen (Table 4). Abdominal pain was associated with increased odds of bacterial pathogen, although the significance was borderline. Nausea was associated with lower odds of a bacterial cause and vomiting for more than 3 days increased the odds of a viral IID, but neither finding was statistically significant.

**Table 4:** Multivariable logistic regression model outputs (Odds Ratio with 95% confidence intervals) for bacterial and viral pathogens, by age group.

Explanatory variable	Odds Ratio (95% confidence intervals)	
	Bacterial	Viral
**Children (<16yrs)**		
Onset in summer	4.37 (1.13–20.07)	0.29 (0.10–0.75)
Diarrhoea but no vomiting	6.69 (2.17–22.21)	0.24 (0.12–0.49)
Fever	4.84 (1.60–17.39)	0.43 (0.21–0.83)
Nausea	0.37 (0.11–1.26)	-
Abdominal pain	3.39 (1.01–13.92)	-
Vomiting lasting >3 days	-	2.59 (0.84–11.40)
**Adults (16-65yrs)**		
Onset in winter	0.31 (0.12–0.79)	4.82 (1.93–12.42)
Onset in spring	0.40 (0.16–0.97)	3.84 (1.58–9.63)
Diarrhoea but no vomiting	3.04 (1.63–5.74)	0.25 (0.14–0.44)
Bloody diarrhoea	6.15 (1.76–26.16)	0.21 (0.05–0.77)
Diarrhoea lasting >3 days	4.90 (2.74–8.95)	0.16 (0.09–0.28)
Loss of appetite	0.34 (0.14–0.79)	3.07 (1.33–7.22)
Fever	1.71 (0.93–3.21)	0.55 (0.29–1.01)
Nausea	0.55 (0.27–1.12)	-
**Elderly (>65yrs)**		
Diarrhoea but no vomiting	3.20 (1.44–7.30)	0.31 (0.14–0.69)
Bloody diarrhoea	7.38 (0.94–161.53)	0.14 (0.01–1.07)
Diarrhoea lasting >3 days	3.31 (1.49–7.59)	0.30 (0.13–0.67)
Loss of appetite	0.43 (0.14–1.22)	2.35 (0.82–7.03)

Adults (16–65 years) had higher odds of a bacterial pathogen if they had diarrhoea but no vomiting (OR 3.04, 95% CI 1.63–5.74), bloody diarrhoea (OR 6.15, 95% CI 1.76–26.16) and diarrhoea lasting more than 3 days (OR 4.90, 95% CI 2.74–8.95). The odds of a bacterial IID were also increased in the presence of fever, and reduced if there was nausea, but these associations were not statistically significant. The odds of a viral pathogen in this age group were increased if onset was in winter (OR 4.82, 95% CI 1.93–12.42) or spring (OR 3.84, 95% CI 1.58–9.63) and there was a loss of appetite (OR 3.07, 95% CI 1.33–7.22).

For elderly adults (>65 years), the odds of bacterial IID were higher if there was diarrhoea but no vomiting (OR 3.20, 95% CI 1.44–7.30), and diarrhoea lasting more than 3 days (OR 3.31, 95% CI 1.49–7.59). These variables were also associated with lower odds of a viral pathogen. Bloody diarrhoea was associated with increased odds of bacterial IID, and loss of appetite with increased odds of viral IID, but neither were significant at the 5% level.

Mild to moderate correlations (phi coefficient <0.5) were identified between some symptoms in the different age groups, such as loss of appetite and nausea. For children, stronger correlations were identified between diarrhoea but no vomiting and nausea (phi = -0.55) and the duration of vomiting and duration of nausea (phi = 0.66). However, there was no evidence of multicollinearity within the regression models (VIF<5) and so all variables were included in the modelling.

## Discussion

This study has identified differences between the symptoms of bacterial and viral IID across different age groups. The odds of bacterial IID in children were increased by onset in the summer months, diarrhoea in the absence of vomiting and fever. These symptoms were also associated with lower odds of a viral pathogen. In adults aged 16–65 years, diarrhoea but no vomiting, bloody diarrhoea and diarrhoea lasting more than 3 days were associated with increased odds of a bacterial organism, whilst onset in the winter or spring and a loss of appetite were positively associated with viral IID. For elderly age groups (>65 years) diarrhoea in the absence of vomiting and diarrhoea lasting more than 3 days were associated with higher odds of bacterial IID and lower odds of a viral cause. Only diarrhoea in the absence of vomiting emerged as a key symptom across all three age groups, associated with increased odds of a bacterial pathogen.

Seasonal patterns of IID are known to vary depending on the individual pathogen, but viral IID is generally associated with peaks in the winter and spring, and bacterial IID with peaks in the summer months [8, 23]. However, this study suggests the association between seasonality and IID varies across age groups. Children experienced an increase in viral IID in the autumn, with an initial peak in late autumn/early winter, and a second larger peak in late winter/early spring. This is consistent with findings that cases of norovirus in children start earlier in the season than cases in adults [20]. Consequently, it was only during summer months that bacterial IID became the more likely cause of infection in a child. Whilst adults showed a more characteristic seasonality, with a large peak of viral IID over winter and spring, this was not the case for elderly adults (>65 years) who showed a less distinct seasonal trend. Furthermore, in the regression model, seasonality was not associated with the odds of either bacterial or viral IID in the elderly age group. Consequently, in our analysis season of onset was not a useful indicator to dissociate between bacterial and viral IID amongst elderly age groups.

Symptom profiling previously identified prolonged diarrhoea (lasting >3 days) as associated with increased odds of a bacterial pathogen [8], and a shorter duration of diarrhoea has typically been associated with viral IID [24]. This study supports those findings for both adult age groups, but not for children. Children were found to have a longer median duration of diarrhoea in viral IID, compared to adult age groups, consistent with findings from other studies that children with norovirus, rotavirus and enteric adenovirus experience protracted diarrhoea [13, 25]. However, this study also identified that children had a similar duration of diarrhoea with both bacterial and viral IID. This is not in keeping with findings of other studies which describe longer mean durations of diarrhoea for bacterial aetiologies in children [25]. One possible explanation is the setting of the study. This study used data from a community cohort and as such will capture milder illness which would otherwise be missed from studies based on hospital admissions or outpatient consultations. As this study identified little difference in the duration of bacterial and viral diarrhoea in children, the duration of diarrhoea may be an unhelpful indicator of underlying cause in children.

The prevalence of reported symptoms may also have an impact on the feasibility of using different symptoms in the development of symptom profiles. Bloody diarrhoea has typically been associated with bacterial IID [8, 24–26], however in this study it was uncommon across all age groups, affecting only 2 children, 22 adults and 7 elderly cases. The low prevalence in this cohort made it a less useful indicator of bacterial IID in our analysis, especially amongst children and elderly. Nausea, headache and abdominal pain were also less common in children and amongst those aged under 2 years there was a larger proportion of missing data for these variables. This is most likely because these symptoms are self-reported, rather than observed, and so cannot be identified in infants and very young children. Consequently, symptom profiles which incorporate these variables may be of limited value for children and adults who are non-verbal and unable to report the presence or absence of these symptoms.

The findings of this study also have implications for the use of epidemiological criteria in outbreak scenarios. Such criteria have used the diarrhoea-to-vomiting ratio, fever-to-vomiting ratio, and the average duration of illness to indicate the likely cause of an IID outbreak [4–6]. However, the finding that symptom profiles vary across age groups could make these epidemiological criteria less applicable to certain settings. For example, the short duration of diarrhoea to indicate norovirus infection may be less appliable to outbreaks in childcare and school settings. Likewise, the use of fever to indicate a bacterial pathogen may not be applicable to elderly care home outbreaks, as fever is less reported in this age group and did not discriminate between bacterial and viral aetiologies. Further work is needed to explore whether these criteria alter between different outbreak settings.

### Limitations

This study provides a valuable comparison of different symptoms of IID across age groups. However, by undertaking a segmented analysis, the resulting sample sizes were reduced, resulting in larger confidence intervals and less certainty around the estimates. To prevent the group sizes becoming too small to allow meaningful analysis, the age bands were split broadly into children, adults and elderly. However, it is recognised that within these groups there may be further symptom variation according to age. This is particularly relevant to the extremes of age, with the very young and very old likely to experience IID differently to others within the broader categories of children and elderly. Unfortunately, the data utilised in this study did not allow this further breakdown by age, but future work should consider exploring these age categories further.

An additional limitation is the use of the broad categories of bacterial and viral IID, rather than developing organism-specific symptom profiles. The analysis was undertaken in this way due to the small number of individual organisms within each age band, which made further breakdown by pathogen unfeasible. However, it should be considered whether the symptom profiles identified in this study could be attributed to different organisms predominating across the three age groups. This is particularly relevant to rotavirus, which disproportionately affects children and typically causes milder illness in adults. Nevertheless, norovirus remained the most common viral pathogen in children and adults alike and *Campylobacter* was the most prevalent bacterial organism in all three age groups. Further study should consider whether pathogen-specific symptom profiles could be developed across the different age groups.

## Conclusion

Symptom profiles for bacterial and viral IID vary across age groups, with evidence of differing seasonality and prevalence of symptoms. Only diarrhoea in the absence of vomiting emerged as a key symptom across all three age groups, associated with increased odds of a bacterial pathogen. These findings have implications for clinicians, public health specialists and epidemiologists who use symptoms to guide presumptive diagnoses in the absence of microbiological confirmation. They may also impact on the use of epidemiological criteria in outbreak situations, as outbreaks in schools and childcare settings may exhibit different symptoms to those in elderly care home settings.

## References

[1] Tam CC, Viviani L, Adak B, Bolton E, Dodds J, Cowden J, et al. The second study of infectious intestinal disease in the community (IID2 study). UK Food Standards Agency, 2012; project no B18021. http://www.food.gov.uk/sites/default/files/media/document/711-1-1393_IID2_FINAL_REPORT.pdf

[2] de Wit MAS, Koopmans MPG, Kortbeek LM, Wannet WJB, Vinjé J, van Leusden F, et al. Sensor, a Population-based Cohort Study on Gastroenteritis in the Netherlands: Incidence and Etiology. Am J Epidemiol. 2001;154(7):666–74. doi: 10.1093/aje/154.7.666 11581101

[3] Humphries RM, Linscott AJ. Laboratory diagnosis of bacterial gastroenteritis. Clin Microbiol Rev. 2015;28(1):3–31. doi: 10.1128/CMR.00073-14 25567220 PMC4284301

[4] Kaplan JE, Gary GW, Baron RC, Singh N, Schonberger LB, Feldman R, et al. Epidemiology of Norwalk gastroenteritis and the role of Norwalk virus in outbreaks of acute nonbacterial gastroenteritis. Ann Intern Med. 1982;96(6 I):756–61. doi: 10.7326/0003-4819-96-6-756 6283977

[5] Hedberg CW, Osterholm MT. Outbreaks of food-borne and waterborne viral gastroenteritis. Clin Microbiol Rev. 1993;6(3):199–210. doi: 10.1128/CMR.6.3.199 8395330 PMC358282

[6] Dalton CB, Mintz ED, Wells JG, Bopp CA, Tauxe R V. Outbreaks of enterotoxigenic Escherichia coli infection in American adults: A clinical and epidemiologic profile. Epidemiol Infect. 1999;123(1):9–16. doi: 10.1017/s0950268899002526 10487636 PMC2810723

[7] Turcios RM, Widdowson MA, Sulka AC, Mead PS, Glass RD. Reevaluation of epidemiological criteria for identifying outbreaks of acute gastroenteritis due to norovirus: United States, 1998–2000. Clin Infect Dis. 2006;42(7):964–9. doi: 10.1086/500940 16511760

[8] Donaldson AL, Clough HE, O’Brien SJ, Harris JP. Symptom profiling for infectious intestinal disease (IID): A secondary data analysis of the IID2 study. Epidemiol Infect. 2019;147. doi: 10.1017/S0950268819001201 31364562 PMC6625207

[9] Baker M, Smith GE, Cooper D, Verlander NQ, Chinemana F, Cotterill S, et al. Early warning and NHS Direct: a role in community surveillance? J Public Health. 2003;25(4):362–8. doi: 10.1093/pubmed/fdg096 14747597

[10] Elliot AJ, Hughes HE, Hughes TC, Locker TE, Shannon T, Heyworth J, et al. Establishing an emergency department syndromic surveillance system to support the London 2012 Olympic and Paralympic Games. Emerg Med J. 2012;29(12):954–60. doi: 10.1136/emermed-2011-200684 22366039

[11] Shah MP, Hall AJ. Norovirus Illnesses in Children and Adolescents. Infect Dis Clin North Am. 2018;32(1):103–18. doi: 10.1016/j.idc.2017.11.004 29406972 PMC6814392

[12] Lindsay L, Wolter J, De Coster I, Van Damme P, Verstraeten T. A decade of norovirus disease risk among older adults in upper-middle and high income countries: A systematic review. BMC Infect Dis. 2015;15(1):1–16. doi: 10.1186/s12879-015-1168-5 26467099 PMC4606836

[13] Harris JP, Iturriza-Gomara M, Allen DJ, Kelly S, O’Brien SJ. Norovirus strain types found within the second infectious intestinal diseases (IID2) study an analysis of norovirus circulating in the community. BMC Infect Dis. 2019;19(1):1–8. 30683063 10.1186/s12879-019-3706-z PMC6346499

[14] Murata T, Katsushima N, Mizuta K, Muraki Y, Hongo S, Matsuzaki Y. Prolonged Norovirus Shedding in Infants ≤6 Months of Age With Gastroenteritis. Pediatr Infect Dis J. 2007;26(1):46–9. doi: 10.1097/01.inf.0000247102.04997.e0 17195705

[15] Mattner F, Sohr D, Heim A, Gastmeier P, Vennema H, Koopmans M. Risk groups for clinical complications of norovirus infections: An outbreak investigation. Clin Microbiol Infect. 2006;12(1):69–74. doi: 10.1111/j.1469-0691.2005.01299.x 16460549

[16] Aoki Y, Suto A, Mizuta K, Ahiko T, Osaka K, Matsuzaki Y. Duration of norovirus excretion and the longitudinal course of viral load in norovirus-infected elderly patients. J Hosp Infect. 2010;75(1):42–6. doi: 10.1016/j.jhin.2009.12.016 20304524

[17] Bernstein DI. Rotavirus Overview. Pediatr Infect Dis J. 2009;28(3 Suppl):S50–3. doi: 10.1097/INF.0b013e3181967bee 19252423

[18] Anderson EJ, Katz BZ, Polin JA, Reddy S, Weinrobe MH, Noskin GA. Rotavirus in adults requiring hospitalization. J Infect. 2012;64(1):89–95. doi: 10.1016/j.jinf.2011.09.003 21939687

[19] Nakajima H, Nakagomi T, Kamisawa T, Sakaki N, Muramoto K, Mikami T, et al. Winter seasonality and rotavirus diarrhoea in adults. Lancet. 2001;357(9272):1950. doi: 10.1016/S0140-6736(00)05086-8 11425422

[20] Bernard H, Hoehne M, Niendorf S, Altmann D, Stark K. Epidemiology of norovirus gastroenteritis in Germany 2001–2009: eight seasons of routine surveillance. Epidemiol Infect. 2014;142(1):63–74. doi: 10.1017/S0950268813000435 23517686 PMC9152553

[21] Tam CC, Rodrigues LC, Viviani L, Dodds JP, Evans MR, Hunter PR, et al. Longitudinal study of infectious intestinal disease in the UK (IID2 study): incidence in the community and presenting to general practice. Gut. 2012;61(1):69–77. doi: 10.1136/gut.2011.238386 21708822 PMC3230829

[22] R: The R Project for Statistical Computing. http://www.r-project.org/

[23] Wiegering V, Kaiser J, Tappe D, Weißbrich B, Morbach H, Girschick HJ. Gastroenteritis in childhood: A retrospective study of 650 hospitalized pediatric patients. Int J Infect Dis. 2011;15(6):e401–7. doi: 10.1016/j.ijid.2011.02.006 21489842

[24] De Wit MAS, Koopmans MPG, Kortbeek LM, Van Leeuwen NJ, Vinjé J, Van Duynhoven YTHP. Etiology of gastroenteritis in sentinel general practices in The Netherlands. Clin Infect Dis. 2001;33(3):280–8. doi: 10.1086/321875 11438890

[25] Uhnoo I, Olding-Stenkvist E, Kreuger A. Clinical features of acute gastroenteritis associated with rotavirus, enteric adenoviruses, and bacteria. Arch Dis Child. 1986;61(8):732–8. doi: 10.1136/adc.61.8.732 3017237 PMC1777930

[26] Liu LJ, Yang YJ, Kuo PH, Wang SM, Liu CC. Diagnostic value of bacterial stool cultures and viral antigen tests based on clinical manifestations of acute gastroenteritis in pediatric patients. Eur J Clin Microbiol Infect Dis. 2005;24(8):559–61. doi: 10.1007/s10096-005-1373-z 16096776

